# Intestinal Microbiota Dysbiosis Promotes Mucosal Barrier Damage and Immune Injury in HIV-Infected Patients

**DOI:** 10.1155/2023/3080969

**Published:** 2023-10-28

**Authors:** Zhaoyi Pan, Nanping Wu, Changzhong Jin

**Affiliations:** ^1^Jinan Microecological Biomedicine Shandong Laboratory, Jinan, China; ^2^State Key Laboratory for Diagnosis and Treatment of Infectious Diseases, National Clinical Research Center for Infectious Diseases, Collaborative Innovation Center for Diagnosis and Treatment of Infectious Diseases, The First Affiliated Hospital, Zhejiang University School of Medicine, Hangzhou, China

## Abstract

The intestinal microbiota is an “invisible organ” in the human body, with diverse components and complex interactions. Homeostasis of the intestinal microbiota plays a pivotal role in maintaining the normal physiological process and regulating immune homeostasis. By reviewing more than one hundred related studies concerning HIV infection and intestinal microbiota from 2011 to 2023, we found that human immunodeficiency virus (HIV) infection can induce intestinal microbiota dysbiosis, which not only worsens clinical symptoms but also promotes the occurrence of post-sequelae symptoms and comorbidities. In the early stage of HIV infection, the intestinal mucosal barrier is damaged and a persistent inflammatory response is induced. Mucosal barrier damage and immune injury play a pivotal role in promoting the post-sequelae symptoms caused by HIV infection. This review summarizes the relationship between dysbiosis of the intestinal microbiota and mucosal barrier damage during HIV infection and discusses the potential mechanisms of intestinal barrier damage induced by intestinal microbiota dysbiosis and inflammation. Exploring these molecular mechanisms might provide new ideas to improve the efficacy of HIV treatment and reduce the incidence of post-sequelae symptoms.

## 1. Introduction

Human immunodeficiency virus (HIV) infection causes acquired immunodeficiency syndrome (AIDS), which is characterized by impaired immune function. HIV infection relies on the interaction of the HIV envelope glycoprotein (gp120/gp41) with the primary receptor CD4 and the coreceptor (chemokine receptor CCR5 or CXCR4) on the cell surface [1]. CD4^+^ T cells are the major targets of HIV infection and depletion of CD4^+^ T cells will impair adaptive immunity functions. In chronic HIV infection, persistent exposure of T cells to high levels of antigen results in T cell exhaustion and finally destroys the immune system [2]. HIV has spread globally for decades and numerous treatments and drugs have been developed to treat HIV infection. Although antiretroviral therapy (ART) is currently the most effective and widely used method to treat AIDS, many patients still face problems of poor peripheral CD4^+^ T cell recovery and obstacles to immune system reconstruction [3–5]. It has been reported that HIV infection can cause intestinal microbiota dysbiosis, which further affects disease progression and the efficacy of ART in patients with AIDS [6–8]. Meanwhile, dysbiosis of the intestinal microbiota not only worsens the inflammatory response but also increases susceptibility to HIV-1 infection [8]. Rebalancing of the intestinal microbiota is also an important treatment strategy for HIV-1 infection. One recent randomized controlled trial on HIV-infected patients demonstrated that fecal microbiota transplantation (FMT) not only improved the richness and evenness of intestinal bacterial taxa but also decreased the intestinal level fatty acid-binding protein (IFABP), a biomarker of intestinal damage that independently predicts mortality [9]. That study revealed that intestinal microbiota manipulation is an effective complementary treatment to alleviate disease progress.

The human gastrointestinal tract contains a large number of microorganisms. At the same time, the gut has evolved important intestinal mucosal immunity to fight against pathogenic microbe infection [10, 11]. The intestinal mucosal immune system includes intestinal endothelial cells, antimicrobial proteins, mucin, and immune cells residing in or scattered throughout the intestinal tract [10]. Furthermore, studies have found that the colonized microbial flora in the gut plays an important role in resistance to other pathogenic microbe infections [12, 13]. Dysbiosis of the intestinal microbiota and intestinal mucosal immune damage are closely related to the occurrence and development of many diseases, such as tumors, cardiovascular diseases, viral infections, and immune diseases [14–18]. Systemic bacteria or lipopolysaccharide (LPS) translocation is increased in patients with intestinal barrier dysfunction [19]. This will not only accelerate multiple opportunistic infections but also worsen inflammation. There is substantial evidence suggesting that the occurrence of Alzheimer's disease (AD) and tumors is closely related to systemic inflammation [20–24]. This indicates that damage to the intestinal barrier could be a pivotal factor that promotes the occurrence of post-sequelae symptoms in HIV-infected patients.

Studies have focused on rebalancing the intestinal microbiome and restoring the mucosal barrier in patients with AIDS to improve the treatment effect [25, 26]. However, the interaction among HIV infection, intestinal microbiota dysbiosis, and mucosal barrier damage is still obscure and requires further exploration. By investigating more than one hundred related studies published in recent decades, we summarize recent research on the interactions among HIV infection, intestinal microbiota dysbiosis, and intestinal mucosal immune damage, and provide a prospective overview for HIV treatment by recovering the intestinal microecological balance and improving intestinal mucosal immunity.

## 2. Intestinal Microecological Dysbiosis in HIV Infection

### 2.1. Gut Dysbacteriosis Induced by HIV Infection

The composition of the gut bacteriome varies significantly by age, socioeconomic factors, geography, and diet [27]. The extent to which HIV infection and the resultant immune compromise alter the gut microbiome remains controversial. However, there is a growing consensus that HIV infection can cause intestinal microecological dysbiosis (Table 1). The diversity of intestinal bacteria indicates microecological stability and the ability to resist pathogenic bacteria invasion. Sequencing-based analysis of bacteria in the intestinal microbiota showed that the alpha diversity of intestinal bacterial was significantly reduced in HIV-infected patients [30, 42]. With the recovery of CD4^+^ T cell counts after ART, the alpha diversity of intestinal bacteria increases accordingly. However, differences in bacterial diversity still exist between HIV-infected patients treated with ART and healthy donors [42].

Using different indexes to evaluate the bacterial diversity will yield different results. Rocafort et al. analyzed the intestinal bacterial diversity of recent and chronically infected patients with HIV using the richness numeric parameters ACE, Chao1, Shannon, and Inverse Simpson [28]. Only the results of ACE showed increased intestinal bacterial diversity in HIV-infected patients, no matter their stages of infection and whether they had been treated with ART or not. The results of the other parameters showed no differences [28]. This increases the difficulty of comparing the results from different studies.

**Figure 1:**
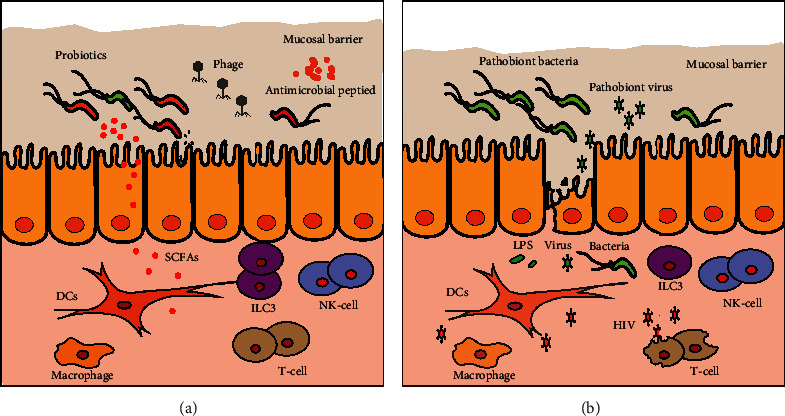
HIV infection damages the intestinal mucosal barrier and induces intestinal microecological dysbiosis. In the early stage of acute HIV infection, CD4^+^ T cells are infected and damaged. In addition, HIV infection induces a significant decrease in ILC3s. This leads to a decrease injunctions between intestinal endothelial cells. Compared with healthy controls (a), HIV infection (b) upregulates the abundance of pro-inflammatory enteric pathogen species and pathobiont viruses, and downregulates the abundance of intestinal microecological homeostasis-maintaining bacterial species. This increases the risk of secondary infection. Decreased levels of antimicrobial peptides and probiotic metabolites will further aggravate dysbiosis of the intestinal microecology.

Dysbiosis of some intestinal microbial genera is very common in HIV infection. Compared with that in healthy controls, the relative abundance of *Prevotella* increased, while the abundance of *Bacteroides*, *Akkermansia*, *Anaerovibrio*, *Bifidobacterium*, and *Clostridium* decreased in the feces of HIV-infected individuals [7, 28, 29]. The dysregulated intestinal microbial genera are different between recent and chronic HIV-1-infected patients. The abundance of *Haemophilus* and *Veillonella* was enriched in the feces of patients recently infected with HIV-1 in their first 6 months [28]. Importantly, the composition of gut microbiota genera is associated with CD4^+^ T cell recovery of HIV-1-infected patients receiving ART. The abundance of intestinal *Faecalibacterium* and *Coprococcus* was decreased in immunological ART responders compared with ART non-responders [7]. Another study reported that *Prevotella*, *Faecalibacterium*, *Bacteroides*, and *Ruminococcus* were enriched in HIV-infected patients with high CD4^+^ T cell counts [31]. Some studies also found that the abundance of the *Desulfovibrionaceae* and *Enterobacteriaceae* families was upregulated in the feces of HIV-infected individuals, while the abundance of *Lachnospiraceae* and *Ruminococcaceae* was downregulated [3, 30, 32, 43]. One recent study reported that the levels of *Bifidobacterium, Collinsella, Faecalibacterium, Oscillospira*, and *Roseburia* were decreased, whereas *Escherichia* was upregulated in HIV-infected patients [33]. Compared with healthy controls, higher abundances of pathogenic bacteria or opportunistic pathogen, as well as lower abundances of butyrate-producing bacteria and bacteria with anti-inflammatory potential, were associated with disease progression in HIV-infected patients [44]. In spite of the different results in different studies, the opportunistic pathogens that can promote an inflammatory response are usually upregulated, and the bacterial species that can produce short-chain fatty acids (SCFAs) and inhibit the inflammatory response are downregulated in HIV-infected patients (Figure 1). For example, the abundance of *Enterobacteriaceae* is upregulated in the gut of patients infected with HIV, in whom it promotes inflammation and its abundance correlates positively with the levels of soluble CD14 (sCD14), interleukin 1 beta (IL-1*β*), and interferon *γ* (IFN*γ*) in the serum [43]. Pathogenic bacterial species might play regulatory roles in reducing CD4^+^ T cell counts in the peripheral blood. These studies demonstrated that HIV infection can destroy the gut microecological homeostasis by upregulating the abundance of pro-inflammatory enteric pathogen species and downregulating the abundance of intestinal anti-inflammatory bacterial species.

### 2.2. Intestinal Mycobiota Dysbiosis Induced by HIV Infection

In addition to bacteria, the intestinal microbiota also comprises fungi, protozoa, archaea, and viruses. A large number of fungi reside in the intestines. As the immune function declines in patients with HIV, the pathogenic fungal communities will cause opportunistic infection. Deaths from invasive fungal infections in patients with HIV account for 50% of global HIV-related mortality [45]. Compared with the comprehensive research about intestinal bacteriome, only a handful of studies regarding the intestinal mycobiome have been published. By analyzing the intestinal fungi in patients with HIV, researchers have found that the intestinal mycobiome is altered in response to HIV infection [34, 35, 37, 46]. The beta diversity of the intestinal mycobiome between healthy controls and patients with HIV is significantly different [37]. Chao1, a richness indicator, was significantly higher in patients with HIV [35]. *Debaryomyceshansenii*, *Candida albicans*, and *Candida parapsilosis* were the most abundant taxa in patients with HIV [35]. The dysbiosis of *Candida albicans* in the intestine can affect digestion and immunity [47]. Besides, one recent study has demonstrated that *Candida albicans* promotes gut dysbiosis and inflammation in patients with HIV [48]. Furthermore, *Candida* spp. is more prevalent in HIV-infected individuals with diarrhea and recent antibiotic treatment than in healthy controls [36]. In addition, another study also found that the abundances of *Candida albicans* and *Candida tropicalis* were higher in patients with HIV [34]. Meanwhile, one study showed that *Aspergillus* was the most abundant genus in the HIV-infected group. The abundance of Thelebolales, Thelebolaceae, and Thlebolus was significantly enriched in HIV-infected individuals [37] (Table 1). The differences in diet and lifestyle of HIV patients might be the reason for the different results. However, there is a consensus that HIV infection could induce intestinal mycobiome dysbiosis.

### 2.3. Gut Virome Dysbiosis in Patients with HIV Infection

The gut virome, which is often referred to as the “dark matter” of the gut microbiome, remains understudied [49]. Compared with intestinal bacterial species, the gut virome always shows greater differences in individuals from different regions, populations, and ages [50]. HIV infection increases the abundance of pathogenic viruses in the human gut, such as adenoviruses [3] (Figure 1). Furthermore, enteric Adenoviridae and Anelloviridae are significantly enriched in HIV-1 infected patients with CD4^+^ T cell counts less than 200 cells/mL [38]. With the recovery of CD4^+^ T cells, the abundance of Adenoviridae decreased significantly [3] (Table 1). Thus, the abundance of Adenoviridae could be a biomarker for ART efficiency. This phenomenon suggests that the increased abundance of virus species associated with intestinal diseases and enteritis in the gut after HIV infection might be related to the development of gastroenteritis and colitis.

Phages, the viruses that can infect and lyse bacteria, can regulate the abundance of bacterial species in the gut. Phages can change intestinal metabolites by regulating the abundance of intestinal bacterial species, and ultimately change the entire intestinal microecology [51]. However, there have been few studies on the relationship between HIV infection and intestinal phages. Whether HIV infection impacts the intestinal phage abundance, thereby regulating the microecology homeostasis, deserves further exploration.

### 2.4. Intestinal Metabolome Disorders Induced by Microbiota Dysbiosis during HIV Infection

HIV infection causes dysbiosis of the intestinal microbiota and further induces intestinal metabolome disorders [52]. HIV infection increases the abundance of gut microbiota that catabolize amino acids through the kynurenic acid metabolic pathway [32, 53], thereby altering microbiome metabolism (Figure 1). Compared with that in healthy controls, tryptophan metabolism was disordered in HIV-infected individuals. Besides, the level of anti-inflammatory metabolites and phosphonoacetate decreased; whereas, phenylethylamine and polyamines were upregulated in HIV-infected patients [44]. The altered gut metabolites can affect physiological processes by entering the circulatory system. There is also an important relationship between the metabolites of the intestinal microbiota and the occurrence of inflammation [54]. HIV infection reduces the abundance of intestinal SCFA-producing bacteria, which leads to decreased intestinal levels of SCFAs [39]. SCFAs can promote the differentiation of intestinal endothelial cells and accelerate the repair of intestinal injury. They also play an important role in regulating the function of immune cells and attenuating the inflammatory response [55]. The HIV-1 elite controllers are a group of people who can spontaneously control virus replication and maintain undetectable plasma viral loads in the absence of ART. Sperk et al. found that the levels of fecal dipeptides in the HIV elite controllers were significantly higher than in HIV progressors and healthy controls. In particular, WG (Tryptophan/Glycine) and VQ (Valine/Glutamine) dipeptides were reported to bind to HIV gp120 and antagonize HIV infection [40]. This suggested that some intestinal metabolites can be used as inhibitors of HIV infection. The intestinal metabolome is also related to neurocognitive disorders in HIV-1 infected patients. Despite the effective inhibition of virus replication by ART, there are still nearly 30% of people with HIV-1 infection that shown neurocognitive dysfunction [56]. Fecal metabolome analysis showed that the contents of bile acids and bioactive lipids were increased, and the levels of vitamin D, terpenoids, and resolvin D1 were decreased, in the feces of HIV-infected patients with cognitive impairment compared with those without cognitive impairment [41] (Table 1). HIV infection can change the microbiome metabolism and further impact the treatment effect and the occurrence of sequelae. However, the complex mechanisms and interactomes among HIV infection, intestinal microbiota dysbiosis, and metabolome disorders remain unclear.

## 3. The Intestinal Mucosal Barrier and HIV Infection

### 3.1. Damage to the Intestinal Mucosal Barrier during HIV Infection

The intestinal mucosal barrier includes immune cells, intestinal endothelial cells, commensal bacteria, and metabolites (antimicrobial molecules and immune-regulating SCFAs). The intestinal mucosal barrier plays an important role in fighting against pathogen micro-infection. Analysis of LPS binding protein (LBP) levels in the serum of SIV-infected rhesus macaques showed that the serum level of LBP was significantly upregulated. Besides, SIV-infected rhesus macaques are more likely to develop gastrointestinal disease [57]. Innate lymphoid cells (ILCs) play an important role in avoiding systemic inflammation caused by intestinal microbe infection. HIV infection can damage the intestinal mucosal barrier by causing a significant decrease in type 3 innate lymphoid cells (ILC3s) in the gut compared with that in healthy controls [58]. A previous study found that damage to the intestinal mucosal barrier is closely related to the occurrence of diarrhea [59]. In the early stage of acute HIV infection, CD4^+^ T cells are infected and experience massive damage. In addition, CD4^+^ T cell subset Th17 cells play an important role in maintaining the tight junction of intestinal endothelial cells and intestinal mucosal immune function (Figure 1). HIV infection induces a reduction in Th17 cells, and damage to the intestinal mucosal immune barrier further reduces its integrity. Subsequently, intestinal microorganisms and microbial products are translocated into the peripheral circulation to induce intestinal inflammation and systemic infections [59–61], such as pneumocystis-pneumonia and cryptococcal meningitis in patients with HIV, which are characterized by increased inflammation in the lung and brain [62, 63]. During the acute stage of primary infection, HIV induces a decline in CD4^+^ T cells, but persistent expansion and over-activation of CD8^+^ T cells. CD8^+^ T cells could suppress HIV replication effectively by releasing the cytotoxic molecules perforin and granzyme B, and secreting antiviral cytokines, including IFN*γ* and tumor necrosis factor alpha (TNF-*α*). However, prolonged HIV antigen stimulation during chronic infection led to CD8^+^T-cell exhaustion and apoptosis [64]. HIV infection can elevate the levels of IL-1*β*, interleukin 6 (IL-6) and TNF-*α* in serum. In addition, the serum level of intestinal fatty acid binding protein (IFABP), a marker of intestinal injury, is also upregulated significantly [65, 66]. This indicates that HIV infection causes damage to the intestinal mucosal barrier and induces an inflammatory response. Furthermore, altered metabolites caused by gut microbiota dysbiosis also promote intestinal mucosal barrier damage [44]. In general, HIV infection disturbs the microbiota community structure, reduces the diversity of the intestinal microbiota, decreases the abundance of probiotics producing immunoregulatory substances, and increases the abundance of pathogenic bacterial species. All these disturbances will destroy the intestinal mucosal immune barrier and increase the risk of pathogenic microbial infection [28, 30, 32, 42].

### 3.2. Damage to the Intestinal Mucosal Barrier Increases the Risk of Secondary Infection

A previous study found that the risk of human papillomavirus (HPV)-related anal cancer was 36 times higher in HIV-infected men than in HIV-negative men. This is mainly caused by HIV infection-induced depletion of tissue-resident memory T cells in the skin and mucosa. Thus, the risk of HPV infection is increased [67]. High molecular weight hyaluronic acid in the extracellular matrix is degraded to low molecular weight hyaluronic acid during acute inflammation. A lack of high molecular weight hyaluronic acid will potentiate HIV infection of CD4^+^ T cells [68]. Intestinal microecological homeostasis plays an important role in resisting pathogenic microbial infection. Furthermore, dysbiosis of the intestinal microecology makes CD4^+^ T cells more susceptible to HIV infection. Studies have revealed that there are differences in the intestinal microbiota between men who have sex with men (MSM) and men who have sex with women (MSW), regardless of the presence or absence of HIV infection. Furthermore, mice transplanted with HIV-negative MSM feces showed stronger CD4^+^ T cell activation than those transplanted with HIV-negative MSW feces, suggesting that the gut microbiota of MSM promotes CD4^+^ T cell activation, making them more susceptible to HIV infection [69]. In addition, altered blood viral composition was found when analyzing the virome in the blood of MSM and HIV-infected individuals. The increased blood viral abundance might be related to damage of the intestinal barrier. Meanwhile, the abundance of phages in blood was decreased and the load of eukaryotic pathogenic viruses, such as anellovirus, pegivirus, and hepatitis B virus, was increased in these groups [70]. Analysis of bacteria in the blood of HIV-infected patients revealed that there was a high similarity between the bacterial composition in the serum and the intestines [71]. By analyzing the circulating bacterial 16S ribosomal DNA (rDNA) in the blood of macaques infected with SIV for eight days. Ericsen et al. found that SIV infection significantly promoted microbial translocation from the intestine to the blood [72]. These studies indicate that HIV/SIV infection causes the shift of intestinal microbial components, which will aggravate infection by pathogenic microorganisms.

## 4. Dysfunction of Intestinal Immune Cells and Inflammatory Response

Immune cells are important to control intestinal pathogenic microbial infection and to maintain intestinal microecological homeostasis. When the intestinal barrier is damaged, pathogenic microbes can infect epithelial cells. Epithelial cells recognize the PAMPs of pathogenic microorganisms through pattern recognition receptors (PRRs), such as TLRs and NOD-like receptors (NLRs), producing high levels of pro-inflammatory cytokines and chemokines. Furthermore, immune cells are recruited to the area of infection to eliminate pathogenic microbes. These immune cells not only regulate the inflammatory response but also promote tissue regeneration and maintain intestinal microecological homeostasis.

### 4.1. Neutrophils

**Figure 2:**
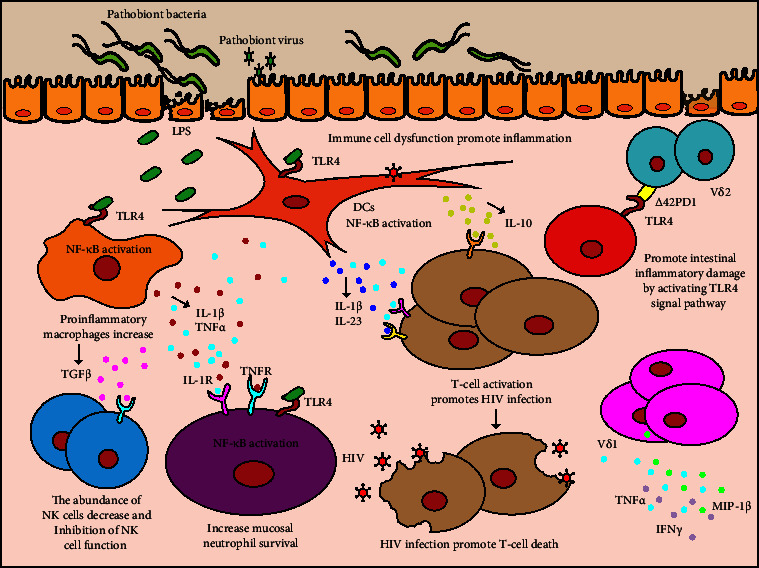
HIV infection induces intestinal mucosal barrier damage and immune cell dysfunction, which aggravates the inflammatory response. HIV infection induces damage to the intestinal mucosal barrier and causes microbial translocation. LPS of the translocated bacteria promotes the survival of neutrophils and the activation of pro-inflammatory macrophages and DCs by activating NF-*κ*B through the TLR4 signaling pathway. Activated macrophages increase the survival of mucosal neutrophils and inhibit the function of NK cells by expressing IL-1*β*, TNF-*α*, and TGF*β*. Activated DCs interact with T cells through CD40/CD40L and secrete cytokines, such as IL-23, IL-1*β*, and IL-10, to further promote the activation of T cells and aggravate the inflammatory response. Δ42PD1^+^ V*δ*2 cells homing to the gut results in small intestinal inflammatory damage by activating the TLR4 signal pathway. The frequencies of V*δ*1^+^ cells producing IFN*γ*, TNF-*α*, and MIP-1*β* were significantly increased in HIV-infected patients.

Neutrophils are the most abundant innate immune cells, playing an important role in resistance to pathogenic microbial infection. After intestinal barrier damage, neutrophils recruited to the site of tissue injury can orchestrate inflammation and tissue repair [73]. However, an abnormal neutrophil response, such as neutrophil infiltration in the colonic mucosa, is closely related to the severity of acute enteritis in inflammatory bowel disease (IBD) [74, 75]. One study found that neutrophil infiltration in the colonic mucosa was increased after HIV infection, which mainly reflected the prolonged life cycle of neutrophils post-HIV infection. That study also revealed that the *Lactobacillus* : *Prevotella* ratio in the intestinal microbiota of HIV-positive patients was decreased, which was related to the survival of neutrophils in the colonic mucosa [76]. *Prevotella copri* has been reported to promote the survival of neutrophils (Figure 2) by activating NF-*κ*B through the TLR4 signaling pathway [77, 78]. In addition, exposure of monocytes to LPS after intestinal mucosal barrier injury promotes the production of the proinflammatory cytokines, TNF-*α* and IL-1*β*, which in turn facilitate the survival of neutrophils (Figure 2) by activating their NF-*κ*B signaling pathway [79]. Gram-negative bacteria containing LPS can promote the survival of neutrophils; however, intestinal probiotic *Lactobacillus*, such as *Lactobacillus plantarum* and *Lactobacillus rhamnosus*, decrease the survival of neutrophils and maintain them at a low level [76]. Thus, the interaction between the gut microbiota and neutrophils might provide a potential therapeutic target to reduce the inflammatory response and improve the efficacy of HIV treatment.

### 4.2. Macrophages

The main function of macrophages in infection is to carry out bacteriophagocytosis (i.e., phagocytosis and digestion) in the innate immune response and to activate lymphocytes or other immune cells in the acquired immune response. They are important for maintaining the integrity and function of the mucosal immune barrier in the intestines [80]. HIV infection is typically characterized by a decrease in the number of circulating monocytes and an increase in proinflammatory macrophages [81]. The exhaustion of intestinal macrophage phagocytic function in HIV-infected patients could promote intestinal microbial translocation, which will result in the accumulation of microbial-derived components and promote HIV-1 disease progression [82]. A recent study found that during HIV infection, the intestinal microbiota plays an important role in dysregulating macrophage function. Compared with treatment by sterile fecal water from HIV-infected individuals with a high CD4^+^ T cell content, treatment with sterile fecal water from HIV-infected individuals with a low CD4^+^ T cell content could decrease phorbol 12-myristate 13-acetate (PMA)-induced differentiation of THP1 cells (immortalized monocytes) (CD14^+^ and CD68^+^), their activation (CD80^+^ and CD86^+^), and their tissue repair capacity (CD206^+^ and IL-10^+^), while the level of cells producing proinflammatory cytokines (e.g., IL-1*β*^+^) was significantly increased [31]. This suggested that gut metabolites from HIV-infected individuals with a low CD4^+^ T cell count can inhibit the differentiation, activation, and tissue repair capacity of macrophages, but promote their inflammatory response. In addition, IL-1*β* produced by pro-inflammatory macrophages promotes neutrophil survival by activating the NF-*κ*B signaling pathway, thereby exacerbating the inflammatory response [79]. Besides, macrophages also play a pivotal role in regulating tissue regeneration [83]. Therefore, dysregulated macrophage function not only promotes an inflammatory response but also impairs the capacity of intestinal repair.

### 4.3. Dendritic Cells (DCs)

DCs are professional antigen-presenting cells in the intestinal mucosa, playing an important role in the control of pathogenic microbial infection and the regulation of intestinal mucosal immune homeostasis [84, 85]. The upregulated pathogenic bacteria in the intestine of HIV-infected patients promote the activation of DCs. CD40 expression on colonic myeloid CD1c^+^ myeloid DCs (mDCs) correlated positively with *Prevotella copri* and *P. stercorea*, and negatively with *Bacteroides acidifaciens*, *Blautia chinkii*, and *Rumminococcus bromii* in the intestine. Besides, the activation of CD1c^+^ mDCs was also associated with the activation of CD4^+^ and CD8^+^ T cells in the colon [86]. Activated CD1c^+^ mDCs interact with T cells through CD40/CD40L and secrete cytokines, such as IL-23, IL-1*β*, and IL-10, to further promote T cell activation (Figure 2) and aggravate the inflammatory response [87–89].

### 4.4. Natural Killer (NK) Cells

HIV infection not only causes chronic inflammation by regulating macrophages and neutrophils through the intestinal microbiota but also causes dysfunction of other immune cells, such as NK cells [90, 91]. NK cells are important to control viral infection. They can identify and kill virus-infected cells by releasing killing factors, such as perforin, NK cytotoxic factor, and TNF-*α* and *β* [92]. They also produce different cytokines to regulate the differentiation and immune response of immune cells [93, 94]. According to the localization of NK cells in the gut, they are divided into two different subsets (intraepithelial lymphocytes and lamina propria). Meanwhile, the frequency of both subsets of NK cells in the gut is downregulated after chronic HIV infection [90]. Furthermore, the levels of serum IFABP and the microbial translocation marker LBP correlate negatively with IFN*γ* production by NK cells. LPS can inhibit the function of NK cells by promoting the production of TGF-*β* by monocytes [91] (Figure 2). The gut microbiota is able to induce the expression of IFN*γ* in NK cells [95]. A recent study demonstrated that IFN*γ* produced by NK cells can be circulated to the brain and inhibit inflammation in the central nervous system [96]. Therefore, promoting the function of NK cells by maintaining intestinal microecological homeostasis and reducing the intestinal chronic inflammatory response might be a promising treatment strategy to improve the efficacy of HIV treatment and reduce the incidence of sequelae.

### 4.5. *γδ* T Cells

*γδ* T cells predominate in the intestinal mucosa and help to maintain gut homeostasis and mucosal immunity. In addition to causing changes in the number of CD4^+^/CD8^+^ T cells, the count of *γδ* T cells is also altered (increased V*δ*1 T cells and decreased V*δ*2 T cells) after HIV infection. The increase in V*δ*1 T cells is similar to the increase in CD8^+^ T cells and the decrease of V*δ*2 T cells is similar to the decrease in CD4^+^ T cells [97]. Therefore, the decrease in the of V*δ*2 : V*δ*1 ratio is an early event similar to the decreased ratio of CD4^+^ : CD8^+^ T cells in early HIV infection [98]. V*δ*1^+^ cells primarily localize within the intestinal mucosa as intraepithelial lymphocytes and help to maintain epithelial function. V*δ*2^+^ cells primarily circulate in blood [99]. Δ42PD1^+^ V*δ*2 cells, a V*δ*2 subset, are significantly upregulated in HIV-infected patients. By expressing gut-homing receptors C-C motif chemokine receptor 9 (CCR9)/CD103 (also known as integrin subunit alpha E), Δ42PD1^+^ V*δ*2 cells home to the gut and induce small intestinal inflammatory damage by activating the TLR4 signaling pathway [100]. It was found that the numbers of V*δ*1^+^ cells producing IFN*γ*, TNF*α*, and macrophage inflammatory protein 1-beta (MIP-1*β*) were significantly increased in HIV-infected patients [101]. Besides, *γδ* T cells play an important role in maintaining the integrity of intestinal epithelial tight junctions to fight against pathogenic microbial infection [102, 103]. Therefore, HIV infection can damage the intestinal barrier and promote inflammation by altering the number and function of *γδ* T cell subsets.

### 4.6. Dysregulated Chronic Inflammation

Chronic inflammation is a key feature of HIV infection. Patients with HIV infection still experience excessive activation of the immune system and chronic inflammation despite ART-induced inhibition of HIV replication [42]. Dysbiosis of the intestinal microbiota is closely related to the occurrence of chronic inflammation [104]. HIV/SIV infection can damage the intestinal mucosal barrier, which promotes the translocation of intestinal microorganisms and aggravates secondary infections. Besides, the abundance of intestinal pathogenic microorganisms is increased after HIV infection. The increased translocation of pathogenic microorganisms and some pathogen-associated molecular patterns (PAMPs), such as LPS, promote the excessive activation of the immune system and chronic inflammation [105, 106]. For example, infection with pathogenic *Fusobacterium nucleatum* could induce intestinal inflammation by promoting the expression of inflammatory factors in the intestinal tract [107]. Compared with HIV infection alone, human herpesvirus 8 (HHV-8) and HIV co-infection can increase the serum levels of IL-4, IL-6, and IL-10 [108]. These results indicate that secondary infections caused by pathogenic bacteria and viruses induced by HIV infection can promote the expression of inflammatory cytokines and the occurrence of chronic inflammation. HIV infection damages the intestinal mucosal barrier and increases the blood levels of intestinal gram-negative bacterial components, such as LPS and LPS-positive bacterial extracellular vesicles, which act as PAMPs to enhance the inflammatory response by activating the Toll-like receptor 4 (TLR4) signaling pathway [19]. In addition, HIV infection decreases the abundance of SCFA-producing bacteria in the gut [109, 110]. SCFAs have important roles in maintaining intestinal homeostasis and the inflammatory response; therefore, a decrease in the abundance of SCFA-producing bacteria will accelerate intestinal inflammation [111]. For example, butyrate can inhibit the inflammatory response and the occurrence of colitis by inhibiting the activation of nuclear factor kappa B (NF-*κ*B) [110].

Chronic inflammation has been reported to contribute to the occurrence of HIV complications [112]. At the same time, chronic inflammation also affects the therapeutic effect of ART. The p38 inhibitor, PH-797804, is an immunosuppressive agent that can effectively inhibit LPS-induced expression of IL-1*β* and TNF-*α* [113]. The combination of PH-797804 and ART significantly alleviated the deterioration of the immune system caused by SIV infection [114]. Intestinal inflammation can destroy the tight junction of intestinal endothelial cells and damage the intestinal mucosal barrier [115], giving rise to translocation of gut microorganisms and aggravation of the inflammatory response. Apolipoprotein A1 (ApoA-I) mimic peptides can bind bioactive esters and LPS in the intestine and inhibit the expression of IL-1*β*, IL-6, C-X3-C motif chemokine ligand 1 (CX3CL1), TNF-*α*, and the intestinal injury marker IFABP, by reducing the expression of ADAM metallopeptidase domain 17 (ADAM17). Compared with ART alone, the combination of ApoA-I mimetic peptides and ART could significantly inhibit intestinal and systemic inflammatory responses in chronic HIV infected humanized murine models [65]. Therefore, maintaining intestinal microecological homeostasis and inhibiting chronic inflammation will be attractive strategies to promote the effect of ART in patients with HIV infection.

## 5. Intestinal Microecological Dysbiosis Promotes HIV Complications

With successful ART, HIV infection has become a chronic disease. Correspondingly, the occurrence of some age-related diseases in patients with HIV has increased, such as cardiovascular diseases and Alzheimer's disease (AD) [116–119]. Even if the viral load is inhibited to an undetectable level using ART, patients with HIV still suffer from increased cardiovascular diseases, such as myocardial infarction, heart failure, stroke, pulmonary hypertension, and sudden cardiac death [116, 117]. Progress has been made in the field of HIV protein-induced cardiovascular and cerebrovascular diseases. For example, HIV Tat has been found to induce the occurrence of cardiovascular and cerebrovascular diseases by regulating apoptosis, autophagy, and the inflammatory response [120]. In recent years, studies have found that the intestinal microbiota is also closely associated with the occurrence of cardiovascular and cerebrovascular diseases during HIV infection. Translocation of intestinal microorganisms through the damaged intestinal mucosal barrier can induce an inflammatory response and promote the occurrence of cardiovascular diseases [121]. Metabolites of intestinal microorganisms also have regulatory effects on the occurrence of cardiovascular diseases. A recent study revealed that 3-hydroxyphenylacetic acid (3-HPA) and 4-hydroxybenzoic acid (4-HBA) can inhibit cell apoptosis through NFE2-related factor 2 (NRF2), thereby protecting cardiac dysfunction after myocardial infarction [122]. Urolithin B, a gut microbiota metabolite, was reported to decrease the myocardial infarct size and attenuate cardiac dysfunction via the p62/Kelch like ECH associated protein 1 (Keap1)/NRF2 signaling pathway [123]. Besides, some microbial metabolites can aggravate cardiovascular disease. L-carnitine, a trimethylamine abundant in red meat, which can be metabolized by microbes into trimethylamine-N-oxide, could accelerate atherosclerosis in mice [124].

Dysbiosis of the intestinal microbiota is reported to be closely related to the occurrence of AD [125–128]. Studies have found that the intestinal microbiota can regulate brain homeostasis through metabolic, endocrinal, neurological, and immune processes, which is referred to as the microbiome-gut-brain axis [126]. HIV infection can promote the occurrence of AD because of the dysbiosis of the intestinal microbiota common in patients with HIV. Although studies have found that the intestinal microbiota *α*-diversity and/or *β*-diversity are significantly different between patients with AD and healthy donors [129–132], there is no consensus that certain bacterial species can promote the occurrence of AD. The reason might be that the altered metabolites caused by microbiota dysbiosis, rather than the microbiota itself, promote the progression of AD. Intestinal metabolites such as gamma-amino butyric acid (GABA), serotonin (5-HT), histamine, dopamine, and SCFAs can regulate the occurrence of AD through the gut-brain axis [128, 133, 134]. In addition, analysis of the gut microbiota and SCFAs in patients with AD showed that these patients had dysbacteriosis and SCFA deficiency in their feces. Meanwhile, levels of blood inflammatory cytokines and intestinal barrier permeability markers (zonulin and alpha-1-antitrypsin) in patients with AD were upregulated significantly [135]. These results indicated that the regulation between the gut and the brain is bidirectional. Recent studies have revealed that HIV infection promotes microbial translocation and immune activation by altering the microbiota [44, 136, 137]. These changes will accelerate opportunistic infections, such as central nervous system (CNS) opportunistic infections, including cerebral toxoplasmosis, progressive multifocal leukoencephalopathy (PML), tuberculous meningitis, cryptococcal meningitis, and cytomegalovirus infection [138, 139]. Opportunistic infections following HIV infection might be an important factor to promote the occurrence of HIV complications. In recent years, significant progress has been made in the treatment and prevention of AD by interfering with the intestinal microbiota. *Lactobacillus* and *Bifidobacterium* can regulate emotion, neurotransmitter transmission, and cognitive impairment by producing GABA and 5-HT [140, 141]. Sodium oligomannate (GV-971) has been reported as a promising drug to treat AD by reshaping the gut microbiome, thereby reducing peripheral and neuro-inflammation [142, 143]. However, there have been few studies on the treatment of AD in HIV-infected patients, especially treatment that interferes with the intestinal microbiota.

## 6. Therapeutic Strategies Associated with Intestinal Microbiota Dysbiosis during HIV Infection

### 6.1. Targeted Therapy by Probiotic Supplementation and Narrow-Spectrum Antibiotics

Probiotic supplementation is an important means to regulate the intestinal microbiota. Probiotics can attenuate inflammation by maintaining intestinal microecological homeostasis [144]. For example, supplementation with SCFA-producing probiotics can promote the production of SCFAs in the intestine. SCFAs not only serve as an energy source for intestinal endothelial cells but also promote their proliferation and differentiation. Thus, SCFA-producing probiotic supplementation can repair the intestinal mucosal barrier and attenuate intestinal inflammation [55]. A previous study reported that supplementation with probiotics can inhibit the translocation of intestinal microorganisms and reduce the blood level of the inflammatory marker sCD14 [145]. The production of IL-22 by ILCs and CD4^+^ T cells plays an important role in maintaining intestinal immune homeostasis. Studies have found that probiotics producing SCFAs can also promote IL-22 production by ILCs and CD4^+^ T cells [146].

Antibiotic treatment is widely used to treat pathogenic bacterial infection. However, the abuse of antibiotics not only leads to the emergence of superbugs and multidrug-resistant bacteria but also causes intestinal microbial dysbiosis [147, 148]. Ridinilazole, a narrow spectrum antibiotic, was reported to be able to treat *Clostridioides difficile* infection with little disruption of the intestinal microbiota [149]. However, the administration of broad-spectrum tetracycline antibiotics (doxycycline and minocycline) significantly decreased microbial diversity and abrogated the recovery of the intestinal microbiota after antibiotic withdrawal [150]. Trimethoprim-sulfamethoxazole has been used as a first-line agent to treat pneumocystis pneumonia in HIV-infected patients with a low CD4^+^ T cell count [151]. Continuous trimethoprim-sulfamethoxazole treatment is reported to have a less disturbing effect on the gut microbiota [152]. Thus, narrow-spectrum antibiotics can be used as intestinal microbiota modulators for targeted therapy with reduced dysbiosis of the gut microbiota.

### 6.2. Fecal Microbiota Transplantation (FMT) and Fecal Virome Transplantation (FVT)

A recent randomized controlled trial found that the intestinal microbiota alpha diversity was significantly increased and the expression of intestinal injury marker IFABP was downregulated after FMT in patients with HIV. The study showed that FMT significantly improved intestinal microbiota dysbiosis and alleviated intestinal damage during HIV infection [9]. Rebalancing the intestinal microbiota could be an effective method to improve HIV treatment. In addition, FVT using the viral component from the gut viral community, in which bacteriophages are dominant, is a very promising therapy to treat pathogenic bacteria infection and might be an alternative treatment to FMT. Significant progress has been made in using bacteriophages to regulate the intestinal microbiota and to treat IBD, pathogenic bacterial infections, and alcoholic liver disease [153–155]. However, using bacteriophages targeting pathogenic bacteria to treat chronic inflammation in patients with HIV has not been reported. Therefore, exploring the gut virome of patients with HIV and screening for phages targeting intestinal pathogenic bacteria to treat chronic inflammation in these patients could provide new ideas and treatment options for the clinical treatment of HIV.

### 6.3. Personalized Dietary Regulation

Different diets can lead to different intestinal microbiotas. A low-fat, high-fiber diet is considered to be beneficial to intestinal microbiota-metabolite modulation. A study found that although high-fiber diets did not change the gut microbial diversity, the levels of microbiome-encoded glycan-degrading carbohydrate active enzymes (CAZymes) were increased [156]. Meanwhile, a high-fermented-food diet could increase the gut microbial diversity, reduce the levels inflammatory cytokines (including nerve growth factor (NGF), LIF receptor subunit alpha (LIF-R), IL-6, IL-10, IL-12b, vascular endothelial growth factor A (VEGFA), matrix metalloproteinase 10 (MMP-10), and chemokine (C-C motif) ligand 9 (CCL9)) in blood and attenuate the activation of inflammatory signals in immune cells (CD4^+^ T cells, CD8^+^ T cells, and B cells) [156]. A ketogenic diet can also change the gut microbial diversity. It reduces the abundance of *Proteobacteria*, increases the abundance of *Akkermansia* in the gut, and decreases the expression of inflammatory cytokines IL-22, IL-17*α*, IL-18, and chemokine C-C motif chemokine ligand 4 (CCL4) in colon tissues [157]. Therefore, personalized dietary regulation can effectively improve intestinal microbial diversity, repair intestinal mucosal immunity, reduce translocation of gut pathogenic microorganisms, and attenuate the inflammatory response.

### 6.4. A Different Voice

Although many approaches have been defined to contribute to the intestinal microecological balance, there are also some studies showing that intestinal microecological modulators are not beneficial to the therapeutic effect in HIV infection. One clinical trial showed that omega-3/6 fatty acids and amino acid supplementation combined with ART did not produce a satisfactory result in the treatment of HIV-positive children [158]. The short nutritional intervention could not attenuate the blood levels of IL-6, IL-7, IP-10, sCD14, and IFABP significantly [158]. In addition, another study found that probiotic supplementation did not promote SIV/HIV-specific T cell and antibody responses induced by HIV/SIV vaccination in rhesus macaques [159]. The reason could be that a short nutritional intervention with prebiotics, probiotics, essential amino acids, or oligonutrient supplementation is not sufficiently effective to change the intestinal microflora. Besides, only 24 children from Spain were recruited in the clinical trial with omega-3/6 fatty acids and amino acids combined with ART to attenuate inflammation [158]. The number of included cases, geographical differences, and dietary composition might impact the effects of intestinal microecological modulators. To this end, it is very important to find intestinal microecological modulators that can effectively regulate intestinal microecological homeostasis. Besides, the molecular mechanisms by which the intestinal microbiota affects the immune response and disease occurrence also require further study.

## 7. Conclusions and Prospects

HIV infection can cause intestinal microbiota dysbiosis and the unbalanced intestinal microorganisms in turn exacerbate the severity and progression of HIV-1 infection. The underlying mechanisms by which intestinal microbiota dysbiosis worsens HIV-1 infection are attributed to the translocation of the inherent bacteria or LPS from the damaged intestinal mucosa and the increased pro-inflammatory metabolites produced by the gut microbiota, which will further induce a severe chronic non-specific inflammatory response and dysfunction of immune cells. In addition, research has verified the relationship between intestinal microbiota dysbiosis and post-sequelae symptoms. Treatment strategies, such as probiotic supplementation, narrow-spectrum antibiotics, FMT, and FVT, have been explored to treat the post-sequelae symptoms of HIV infection.

Although the importance and complexity of the intestinal microbiome for HIV-1 infection have been widely recognized by researchers, there are still many mechanisms and effects that are unclear. We suggest that the following aspects should be valued in future studies.

- The intestinal microbiota consists of not only of bacteria but also a variety of viruses and fungi, which can interact with each other directly (such as bacteriophages infecting bacteria) or indirectly (through metabolites or cytokines). The intricate relationship and interaction among intestinal microorganisms will undoubtedly increase the complexity of intestinal microbiota research.
- With the wide use of ART globally, the life span of HIV-infected patients has been largely extended. Non-AIDS-related diseases and post-sequelae symptoms have been hot topics in current clinical research of HIV infection. Therefore, the relationship between intestinal microecology and HIV post-sequelae symptoms and non-AIDS related diseases should be investigated.
- The mechanisms underlying the interaction between intestinal microecological dysbiosis and disease progression of HIV infection are still unclear. Exploring the molecular mechanism of intestinal microecology aggravation/alleviation of disease occurrence and the development of post-sequelae symptoms of HIV infection could provide references for the optimization of clinical disease treatment and drug development. However, limitations of research methods, especially the lack of animal models, have retarded research progress.
- Rebalancing the intestinal microbiome of HIV-infected patients will be another interesting topic. An ideal and promising intestinal microbiota modulator for HIV-infected patients could be a complex that can collectively improve the intestinal microenvironment, enhance the efficacy of HIV treatment, and reduce the incidence of post-sequelae symptoms. On the one hand, in response to the decrease in probiotics in the intestines, probiotics and prebiotics could be supplemented directly. On the other hand, to inhibit the upregulated pathogenic bacteria and viruses in the intestine, narrow-spectrum antibiotic therapy could be used as intestinal microbiota modulators for targeted therapy. Other therapeutic approaches, such as FMT, FVT and phage therapy, provide new therapeutic ideas for future treatment.
- Finally, a notable problem for the present microecology study of HIV-infected patients is that the majority of existing studies have focused on the bacteria in the intestines, while the virome and mycobiome in the intestines of HIV-infected patients have received much less attention. Changes in the profiles of the virome and mycobiome, and the interactions among the bacteriome, virome, and mycobiome are mostly unknown. This “dark matter” of intestinal microbiology represents a “gold mine” for future study.

## Figures and Tables

**Table 1:** Intestinal microecological dysbiosis during HIV infection.

Groups	Alterations	Reference
Bacteriome	*Prevotella* increased; *Bacteroides*, *Akkermansia*, *Anaerovibrio*, *Bifidobacterium*, and *Clostridium* decreased in patients with HIV	[7, 28, 29]
	*Haemophilus* and *Veillonella* were enriched in patients recently infected with HIV-1 in their first 6 months	[28]
	*Faecalibacterium* and *Coprococcus* levels decreased in immunological ART responders compared with ART non-responders	[7]
	Desulfovibrionaceae and enterobacteriaceae were upregulated in HIV-infected individuals, lachnospiraceae and ruminococcaceae were downregulated	[3, 30–32]
	*Bifidobacterium, Collinsella, Faecalibacterium, Oscillospira*, and *Roseburia* levels decreased, whereas *Escherichia* was upregulated in HIV-infected patients	[33]

Mycobiome	*Debaryomyceshansenii*, *Candida albicans*, *Candida tropicalis*, and *Candida parapsilosis* were the most abundant taxa in patients with HIV	[34, 35]
	*Candida* spp. are more prevalent in HIV-infected individuals with diarrhea and recent antibiotic treatment	[36]
	*Aspergillus* was the most abundant genus (49.92%) in the HIV-infected group	[37]

Virome	HIV infection increase the abundance of adenoviruses	[3]
	*Adenoviridae* and *Anelloviridae* are significantly enriched in HIV-1-infected patients with CD4^+^ T cell less than 200 cells/mL	[38]

Metabolome	HIV infection decreases intestinal SCFA levels	[39]
	WG and VQ dipeptide levels were significantly higher in the HIV elite controllers	[40]
	Bile acids and bioactive lipids increased; vitamin D, terpenoids, and resolvin D1 decreased in the feces of HIV-infected patients with cognitive impairment	[41]

## Data Availability

All available data have been included in the manuscript.

## References

[1] Chen B. (2019). Molecular mechanism of HIV-1 entry. *Trends in Microbiology*.

[2] Fenwick C., Joo V., Jacquier P. (2019). T-cell exhaustion in HIV infection. *Immunological Reviews*.

[3] Monaco C. L., Gootenberg D. B., Zhao G. (2016). Altered virome and bacterial microbiome in human immunodeficiency virus-associated acquired immunodeficiency syndrome. *Cell Host and Microbe*.

[4] Ejigu Y., Magnus J. H., Sundby J., Magnus M. (2019). Health outcomes of asymptomatic HIV-infected pregnant women initiating antiretroviral therapy at different baseline CD4 counts in Ethiopia. *International Journal of Infectious Diseases*.

[5] Ford N., Meintjes G., Pozniak A. (2015). The future role of CD4 cell count for monitoring antiretroviral therapy. *The Lancet Infectious Diseases*.

[6] Lozupone C. A., Rhodes M. E., Neff C. P., Fontenot A. P., Campbell T. B., Palmer B. E. (2014). HIV-induced alteration in gut microbiota: driving factors, consequences, and effects of antiretroviral therapy. *Gut Microbes*.

[7] Lu W., Feng Y., Jing F. (2018). Association between gut microbiota and CD4 recovery in HIV-1 infected patients. *Frontiers in Microbiology*.

[8] Chen Y., Lin H., Cole M. (2021). Signature changes in gut microbiome are associated with increased susceptibility to HIV-1 infection in MSM. *Microbiome*.

[9] Serrano-Villar S., Talavera-Rodriguez A., Gosalbes M. J. (2021). Fecal microbiota transplantation in HIV: a pilot placebo-controlled study. *Nature Communications*.

[10] Perez-Lopez A., Behnsen J., Nuccio S. P., Raffatellu M. (2016). Mucosal immunity to pathogenic intestinal bacteria. *Nature Reviews Immunology*.

[11] Zou J., Liu C., Jiang S., Qian D., Duan J. (2021). Cross talk between gut microbiota and intestinal mucosal immunity in the development of ulcerative colitis. *Infection and Immunity*.

[12] Lin S., Mukherjee S., Li J., Hou W., Pan C., Liu J. (2021). Mucosal immunity-mediated modulation of the gut microbiome by oral delivery of probiotics into Peyer’s patches. *Science Advances*.

[13] Kamada N., Seo S. U., Chen G. Y., Nunez G. (2013). Role of the gut microbiota in immunity and inflammatory disease. *Nature Reviews Immunology*.

[14] Garrett W. S. (2015). Cancer and the microbiota. *Science*.

[15] Witkowski M., Weeks T. L., Hazen S. L. (2020). Gut microbiota and cardiovascular disease. *Circulation Research*.

[16] Penninger J. M., Grant M. B., Sung J. J. Y. (2021). The role of angiotensin converting enzyme 2 in modulating gut microbiota, intestinal inflammation, and coronavirus infection. *Gastroenterology*.

[17] Ruff W. E., Greiling T. M., Kriegel M. A. (2020). Host-microbiota interactions in immune-mediated diseases. *Nature Reviews Microbiology*.

[18] Budden K. F., Shukla S. D., Rehman S. F. (2019). Functional effects of the microbiota in chronic respiratory disease. *The Lancet Respiratory Medicine*.

[19] Tulkens J., Vergauwen G., Van Deun J. (2020). Increased levels of systemic LPS-positive bacterial extracellular vesicles in patients with intestinal barrier dysfunction. *Gut*.

[20] Heneka M. T., Carson M. J., Khoury J. E. (2015). Neuroinflammation in Alzheimer’s disease. *The Lancet Neurology*.

[21] Newcombe E. A., Camats-Perna J., Silva M. L., Valmas N., Huat T. J., Medeiros R. (2018). Inflammation: the link between comorbidities, genetics, and Alzheimer’s disease. *Journal of Neuroinflammation*.

[22] Holmes C. (2013). Review: systemic inflammation and Alzheimer’s disease. *Neuropathology and Applied Neurobiology*.

[23] Greten F. R., Grivennikov S. I. (2019). Inflammation and cancer: triggers, mechanisms, and consequences. *Immunity*.

[24] Hibino S., Kawazoe T., Kasahara H. (2021). Inflammation-induced tumorigenesis and metastasis. *International Journal of Molecular Sciences*.

[25] Crakes K. R., Santos Rocha C., Grishina I. (2019). PPAR*α*-targeted mitochondrial bioenergetics mediate repair of intestinal barriers at the host–microbe intersection during SIV infection. *Proceedings of the National Academy of Sciences of the U S A*.

[26] Irvine S. L., Hummelen R., Hekmat S., W N Looman C., Habbema J. D. F., Reid G. (2010). Probiotic yogurt consumption is associated with an increase of CD4 count among people living with HIV/AIDS. *Journal of Clinical Gastroenterology*.

[27] Liu J., Williams B., Frank D., Dillon S. M., Wilson C. C., Landay A. L. (2017). Inside out: HIV, the gut microbiome, and the mucosal immune system. *The Journal of Immunology*.

[28] Rocafort M., Noguera-Julian M., Rivera J. (2019). Evolution of the gut microbiome following acute HIV-1 infection. *Microbiome*.

[29] Meng J., Tao J., Abu Y. (2023). HIV-positive patients on antiretroviral therapy have an altered mucosal intestinal but not oral microbiome. *Microbiology Spectrum*.

[30] Vujkovic-Cvijin I., Sortino O., Verheij E. (2020). HIV-associated gut dysbiosis is independent of sexual practice and correlates with noncommunicable diseases. *Nature Communications*.

[31] Shenoy M. K., Fadrosh D. W., Lin D. L. (2019). Gut microbiota in HIV-pneumonia patients is related to peripheral CD4 counts, lung microbiota, and in vitro macrophage dysfunction. *Microbiome*.

[32] Vujkovic-Cvijin I., Dunham R. M., Iwai S. (2013). Dysbiosis of the gut microbiota is associated with HIV disease progression and tryptophan catabolism. *Science Translational Medicine*.

[33] Zhao H., Feng A., Luo D. (2023). Altered gut microbiota is associated with different immunologic responses to antiretroviral therapy in HIV-infected men who have sex with men. *Journal of Medical Virology*.

[34] Hamad I., Abou Abdallah R., Ravaux I. (2018). Metabarcoding analysis of eukaryotic microbiota in the gut of HIV-infected patients. *PLoS One*.

[35] Gosalbes M. J., Jimenez-Hernandez N., Moreno E. (2022). Interactions among the mycobiome, bacteriome, inflammation, and diet in people living with HIV. *Gut Microbes*.

[36] Gouba N., Drancourt M. (2015). Digestive tract mycobiota: a source of infection. *Medecine et Maladies Infectieuses*.

[37] Yin Y., Tuohutaerbieke M., Feng C. (2022). Characterization of the intestinal fungal microbiome in HIV and HCV mono-infected or Co-infected patients. *Viruses*.

[38] Monaco C. L. (2022). HIV, AIDS, and the virome: gut reactions. *Cell Host and Microbe*.

[39] El-Far M., Durand M., Turcotte I. (2021). Upregulated IL-32 expression and reduced gut short chain fatty acid caproic acid in people living with HIV with subclinical atherosclerosis. *Frontiers in Immunology*.

[40] Sperk M., Ambikan A. T., Ray S. (2021). Fecal metabolome signature in the HIV-1 elite control phenotype: enrichment of dipeptides acts as an HIV-1 antagonist but a Prevotella agonist. *Journal of Virology*.

[41] Dong R., Lin H., Chen X. (2021). Gut microbiota and fecal metabolites associated with neurocognitive impairment in HIV-infected population. *Frontiers in Cellular and Infection Microbiology*.

[42] Ishizaka A., Koga M., Mizutani T. (2021). Unique gut microbiome in HIV patients on antiretroviral therapy (ART) suggests association with chronic inflammation. *Microbiology Spectrum*.

[43] Dinh D. M., Volpe G. E., Duffalo C. (2015). Intestinal microbiota, microbial translocation, and systemic inflammation in chronic HIV infection. *The Journal of Infectious Diseases*.

[44] Zhang Y., Xie Z., Zhou J. (2022). The altered metabolites contributed by dysbiosis of gut microbiota are associated with microbial translocation and immune activation during HIV infection. *Frontiers in Immunology*.

[45] Lockhart S. R., Guarner J. (2019). Emerging and reemerging fungal infections. *Seminars in Diagnostic Pathology*.

[46] Annavajhala M. K., Khan S. D., Sullivan S. B. (2020). Oral and gut microbial diversity and immune regulation in patients with HIV on antiretroviral therapy. *mSphere*.

[47] Musumeci S., Coen M., Leidi A., Schrenzel J. (2022). The human gut mycobiome and the specific role of Candida albicans: where do we stand, as clinicians?. *Clinical Microbiology and Infection*.

[48] Zaongo S. D., Ouyang J., Isnard S. (2023). Candida albicans can foster gut dysbiosis and systemic inflammation during HIV infection. *Gut Microbes*.

[49] Neurath M. F., Uberla K., Ng S. C. (2021). Gut as viral reservoir: lessons from gut viromes, HIV and COVID-19. *Gut*.

[50] Zuo T., Sun Y., Wan Y. (2020). Human-gut-DNA virome variations across geography, ethnicity, and urbanization. *Cell Host and Microbe*.

[51] Hsu B. B., Gibson T. E., Yeliseyev V. (2019). Dynamic modulation of the gut microbiota and metabolome by bacteriophages in a mouse model. *Cell Host and Microbe*.

[52] Taylor B. C., Weldon K. C., Ellis R. J. (2020). Depression in individuals coinfected with HIV and HCV is associated with systematic differences in the gut microbiome and metabolome. *mSystems*.

[53] Qi Q., Hua S., Clish C. B. (2018). Plasma tryptophan-kynurenine metabolites are altered in human immunodeficiency virus infection and associated with progression of carotid artery atherosclerosis. *Clinical Infectious Diseases*.

[54] Vazquez-Castellanos J. F., Serrano-Villar S., Jimenez-Hernandez N. (2018). Interplay between gut microbiota metabolism and inflammation in HIV infection. *The ISME Journal*.

[55] Martin-Gallausiaux C., Marinelli L., Blottiere H. M., Larraufie P., Lapaque N. (2021). SCFA: mechanisms and functional importance in the gut. *Proceedings of the Nutrition Society*.

[56] Heaton R. K., Franklin D. R., Ellis R. J. (2011). HIV-associated neurocognitive disorders before and during the era of combination antiretroviral therapy: differences in rates, nature, and predictors. *Journal of NeuroVirology*.

[57] Handley S. A., Desai C., Zhao G. (2016). SIV infection-mediated changes in gastrointestinal bacterial microbiome and virome are associated with immunodeficiency and prevented by vaccination. *Cell Host and Microbe*.

[58] Wang Y., Lifshitz L., Gellatly K. (2020). HIV-1-induced cytokines deplete homeostatic innate lymphoid cells and expand TCF7-dependent memory NK cells. *Nature Immunology*.

[59] Logan C., Beadsworth M. B., Beeching N. J. (2016). HIV and diarrhoea: what is new?. *Current Opinion in Infectious Diseases*.

[60] George M. D., Asmuth D. M. (2014). Mucosal immunity in HIV infection: what can be done to restore gastrointestinal-associated lymphoid tissue function?. *Current Opinion in Infectious Diseases*.

[61] Godfrey C., Bremer A., Alba D. (2019). Obesity and fat metabolism in human immunodeficiency virus-infected individuals: immunopathogenic mechanisms and clinical implications. *The Journal of Infectious Diseases*.

[62] Benfield T. L., Prento P., Junge J., Vestbo J., Lundgren J. D. (1997). Alveolar damage in AIDS-related Pneumocystis carinii pneumonia. *Chest*.

[63] Tenforde M. W., Scriven J. E., Harrison T. S., Jarvis J. N. (2017). Immune correlates of HIV-associated cryptococcal meningitis. *PLoS Pathogens*.

[64] Zhang C., Hu W., Jin J. H. (2020). The role of CD8 T cells in controlling HIV beyond the antigen-specific face. *HIV Medicine*.

[65] Daskou M., Mu W., Sharma M. (2022). ApoA-I mimetics reduce systemic and gut inflammation in chronic treated HIV. *PLoS Pathogens*.

[66] Mu W., Sharma M., Heymans R. (2021). Apolipoprotein A-I mimetics attenuate macrophage activation in chronic treated HIV. *AIDS*.

[67] Saluzzo S., Pandey R. V., Gail L. M. (2021). Delayed antiretroviral therapy in HIV-infected individuals leads to irreversible depletion of skin- and mucosa-resident memory T cells. *Immunity*.

[68] Egedal J. H., Xie G., Packard T. A. (2021). Hyaluronic acid is a negative regulator of mucosal fibroblast-mediated enhancement of HIV infection. *Mucosal Immunology*.

[69] Li S. X., Sen S., Schneider J. M. (2019). Gut microbiota from high-risk men who have sex with men drive immune activation in gnotobiotic mice and in vitro HIV infection. *PLoS Pathogens*.

[70] Liu K., Li Y., Xu R. (2021). HIV-1 infection alters the viral composition of plasma in men who have sex with men. *mSphere*.

[71] Li S. K., Leung R. K., Guo H. X. (2012). Detection and identification of plasma bacterial and viral elements in HIV/AIDS patients in comparison to healthy adults. *Clinical Microbiology and Infection*.

[72] Ericsen A. J., Lauck M., Mohns M. S. (2016). Microbial translocation and inflammation occur in hyperacute immunodeficiency virus infection and compromise host control of virus replication. *PLoS Pathogens*.

[73] Kvedaraite E. (2021). Neutrophil-T cell crosstalk in inflammatory bowel disease. *Immunology*.

[74] Muthas D., Reznichenko A., Balendran C. A. (2017). Neutrophils in ulcerative colitis: a review of selected biomarkers and their potential therapeutic implications. *Scandinavian Journal of Gastroenterology*.

[75] Bressenot A., Salleron J., Bastien C., Danese S., Boulagnon-Rombi C., Peyrin-Biroulet L. (2015). Comparing histological activity indexes in UC. *Gut*.

[76] Hensley-Mcbain T., Wu M. C., Manuzak J. A. (2019). Increased mucosal neutrophil survival is associated with altered microbiota in HIV infection. *PLoS Pathogens*.

[77] Vatanen T., Kostic A. D., D’hennezel E. (2016). Variation in microbiome LPS immunogenicity contributes to autoimmunity in humans. *Cell*.

[78] Ward C., Chilvers E. R., Lawson M. F. (1999). NF-*κ*B activation is a critical regulator of human granulocyte apoptosis in vitro. *Journal of Biological Chemistry*.

[79] Sabroe I., Prince L. R., Jones E. C. (2003). Selective roles for Toll-like receptor (TLR)2 and TLR4 in the regulation of neutrophil activation and life span. *The Journal of Immunology*.

[80] Chikina A. S., Nadalin F., Maurin M. (2020). Macrophages maintain epithelium integrity by limiting fungal product absorption. *Cell*.

[81] Allers K., Fehr M., Conrad K. (2014). Macrophages accumulate in the gut mucosa of untreated HIV-infected patients. *Journal of Infectious Diseases*.

[82] Santinelli L., Rossi G., Gioacchini G. (2023). The crosstalk between gut barrier impairment, mitochondrial dysfunction, and microbiota alterations in people living with HIV. *Journal of Medical Virology*.

[83] Vannella K. M., Wynn T. A. (2017). Mechanisms of organ injury and repair by macrophages. *Annual Review of Physiology*.

[84] Luciani C., Hager F. T., Cerovic V., Lelouard H. (2022). Dendritic cell functions in the inductive and effector sites of intestinal immunity. *Mucosal Immunology*.

[85] Stagg A. J. (2018). Intestinal dendritic cells in health and gut inflammation. *Frontiers in Immunology*.

[86] Dillon S. M., Lee E. J., Kotter C. V. (2014). An altered intestinal mucosal microbiome in HIV-1 infection is associated with mucosal and systemic immune activation and endotoxemia. *Mucosal Immunology*.

[87] Dillon S. M., Manuzak J. A., Leone A. K. (2012). HIV-1 infection of human intestinal lamina propria CD4+ T cells in vitro is enhanced by exposure to commensal *Escherichia coli*. *The Journal of Immunology*.

[88] Steele A. K., Lee E. J., Manuzak J. A. (2014). Microbial exposure alters HIV-1-induced mucosal CD4+ T cell death pathways Ex vivo. *Retrovirology*.

[89] Dillon S. M., Lee E. J., Kotter C. V. (2016). Gut dendritic cell activation links an altered colonic microbiome to mucosal and systemic T-cell activation in untreated HIV-1 infection. *Mucosal Immunology*.

[90] Sips M., Sciaranghella G., Diefenbach T. (2012). Altered distribution of mucosal NK cells during HIV infection. *Mucosal Immunology*.

[91] Tovinh M., Horr G., Hoffmeister C. (2022). HIV-associated microbial translocation may affect cytokine production of CD56bright NK cells via stimulation of monocytes. *The Journal of Infectious Diseases*.

[92] Rabinowich H., Sedlmayr P., Herberman R. B., Whiteside T. L. (1993). Response of human NK cells to IL-6 alterations of the cell surface phenotype, adhesion to fibronectin and laminin, and tumor necrosis factor-alpha/beta secretion. *The Journal of Immunology*.

[93] Vivier E., Raulet D. H., Moretta A. (2011). Innate or adaptive immunity? The example of natural killer cells. *Science*.

[94] Li H., Zhai N., Wang Z. (2018). Regulatory NK cells mediated between immunosuppressive monocytes and dysfunctional T cells in chronic HBV infection. *Gut*.

[95] Thiemann S., Smit N., Roy U. (2017). Enhancement of IFN*γ* production by distinct commensals ameliorates salmonella-induced disease. *Cell Host and Microbe*.

[96] Sanmarco L. M., Wheeler M. A., Gutierrez-Vazquez C. (2021). Gut-licensed IFN*γ*+ NK cells drive LAMP1+TRAIL+ anti-inflammatory astrocytes. *Nature*.

[97] Li H., Chaudry S., Poonia B., Shao Y., Pauza C. D. (2013). Depletion and dysfunction of V*γ*2V*δ*2 T cells in HIV disease: mechanisms, impacts and therapeutic implications. *Cellular and Molecular Immunology*.

[98] Pauza C. D., Poonia B., Li H., Cairo C., Chaudhry S. (2014). Γ*δ* T cells in HIV disease: past, present, and future. *Frontiers in Immunology*.

[99] Vantourout P., Hayday A. (2013). Six-of-the-best: unique contributions of *γδ* T cells to immunology. *Nature Reviews Immunology*.

[100] Cheung A. K. L., Kwok H. Y., Huang Y. (2017). Gut-homing Δ42PD1+V*δ*2 T cells promote innate mucosal damage via TLR4 during acute HIV type 1 infection. *Nat Microbiol*.

[101] Olson G. S., Moore S. W., Richter J. M. (2018). Increased frequency of systemic pro-inflammatory V*δ*1+ *γδ* T cells in HIV elite controllers correlates with gut viral load. *Scientific Reports*.

[102] Dalton J. E., Cruickshank S. M., Egan C. E. (2006). Intraepithelial *γδ*+ lymphocytes maintain the integrity of intestinal epithelial tight junctions in response to infection. *Gastroenterology*.

[103] Nielsen M. M., Witherden D. A., Havran W. L. (2017). *γδ* T cells in homeostasis and host defence of epithelial barrier tissues. *Nature Reviews Immunology*.

[104] Rizzetto L., Fava F., Tuohy K. M., Selmi C. (2018). Connecting the immune system, systemic chronic inflammation and the gut microbiome: the role of sex. *Journal of Autoimmunity*.

[105] Bell L. C. K., Noursadeghi M. (2018). Pathogenesis of HIV-1 and *Mycobacterium tuberculosis* co-infection. *Nature Reviews Microbiology*.

[106] Zaongo S. D., Ouyang J., Chen Y., Jiao Y. M., Wu H., Chen Y. (2022). HIV infection predisposes to increased chances of HBV infection: current understanding of the mechanisms favoring HBV infection at each clinical stage of HIV infection. *Frontiers in Immunology*.

[107] Engevik M. A., Danhof H. A., Ruan W. (2021). Fusobacterium nucleatum secretes outer membrane vesicles and promotes intestinal inflammation. *mBio*.

[108] Silva D. M. D., Goncales J. P., Silva Junior J. V. J. (2021). Evaluation of IL-2, IL-4, IL-6, IL-10, TNF-*α*, and IFN-*γ* cytokines in HIV/HHV-8 coinfection. *Journal of Medical Virology*.

[109] Kelly C. J., Zheng L., Campbell E. L. (2015). Crosstalk between microbiota-derived short-chain fatty acids and intestinal epithelial HIF augments tissue barrier function. *Cell Host and Microbe*.

[110] Inan M. S., Rasoulpour R. J., Yin L., Hubbard A. K., Rosenberg D. W., Giardina C. (2000). The luminal short-chain fatty acid butyrate modulates NF-*κ*B activity in a human colonic epithelial cell line. *Gastroenterology*.

[111] Parada Venegas D., De La Fuente M. K., Landskron G. (2019). Short chain fatty acids (SCFAs)-Mediated gut epithelial and immune regulation and its relevance for inflammatory bowel diseases. *Frontiers in Immunology*.

[112] Peterson T. E., Baker J. V. (2019). Assessing inflammation and its role in comorbidities among persons living with HIV. *Current Opinion in Infectious Diseases*.

[113] Hope H. R., Anderson G. D., Burnette B. L. (2009). Anti-inflammatory properties of a novel N-phenyl pyridinone inhibitor of p38 mitogen-activated protein kinase: preclinical-to-clinical translation. *Journal of Pharmacology and Experimental Therapeutics*.

[114] Chaudhary O., Narayan V., Lelis F. (2018). Inhibition of p38 MAPK in combination with ART reduces SIV-induced immune activation and provides additional protection from immune system deterioration. *PLoS Pathogens*.

[115] Freour T., Jarry A., Bach-Ngohou K. (2009). TACE inhibition amplifies TNF-alpha-mediated colonic epithelial barrier disruption. *International Journal of Molecular Medicine*.

[116] Feinstein M. J., Hsue P. Y., Benjamin L. A. (2019). Characteristics, prevention, and management of cardiovascular disease in people living with HIV: a scientific statement from the American heart association. *Circulation*.

[117] Mclaughlin M. M., Ma Y., Scherzer R. (2020). Association of viral persistence and atherosclerosis in adults with treated HIV infection. *JAMA Network Open*.

[118] Jha N. K., Sharma A., Jha S. K. (2020). Alzheimer’s disease-like perturbations in HIV-mediated neuronal dysfunctions: understanding mechanisms and developing therapeutic strategies. *Open Biol*.

[119] Vance D. E., Brew B. J. (2021). HIV neurocognitive impairment and Alzheimer’s disease: sniffing out the difference. *AIDS*.

[120] Jiang Y., Chai L., Fasae M. B., Bai Y. (2018). The role of HIV Tat protein in HIV-related cardiovascular diseases. *Journal of Translational Medicine*.

[121] Zhou X., Li J., Guo J. (2018). Gut-dependent microbial translocation induces inflammation and cardiovascular events after ST-elevation myocardial infarction. *Microbiome*.

[122] Zhou Q., Deng J., Pan X. (2022). Gut microbiome mediates the protective effects of exercise after myocardial infarction. *Microbiome*.

[123] Zheng D., Liu Z., Zhou Y. (2020). Urolithin B, a gut microbiota metabolite, protects against myocardial ischemia/reperfusion injury via p62/Keap1/Nrf2 signaling pathway. *Pharmacological Research*.

[124] Koeth R. A., Wang Z., Levison B. S. (2013). Intestinal microbiota metabolism of L-carnitine, a nutrient in red meat, promotes atherosclerosis. *Nature Medicine*.

[125] Megur A., Baltriukiene D., Bukelskiene V., Burokas A. (2020). The microbiota-gut-brain Axis and Alzheimer’s disease: neuroinflammation is to blame?. *Nutrients*.

[126] Liu S., Gao J., Zhu M., Liu K., Zhang H. L. (2020). Gut microbiota and dysbiosis in Alzheimer’s disease: implications for pathogenesis and treatment. *Molecular Neurobiology*.

[127] Chen C., Liao J., Xia Y. (2022). Gut microbiota regulate Alzheimer’s disease pathologies and cognitive disorders via PUFA-associated neuroinflammation. *Gut*.

[128] Wang Q., Luo Y., Ray Chaudhuri K., Reynolds R., Tan E. K., Pettersson S. (2021). The role of gut dysbiosis in Parkinson’s disease: mechanistic insights and therapeutic options. *Brain*.

[129] Barichella M., Severgnini M., Cilia R. (2019). Unraveling gut microbiota in Parkinson’s disease and atypical parkinsonism. *Movement Disorders*.

[130] Li C., Cui L., Yang Y. (2019). Gut microbiota differs between Parkinson’s disease patients and healthy controls in northeast China. *Frontiers in Molecular Neuroscience*.

[131] Qian Y., Yang X., Xu S. (2018). Alteration of the fecal microbiota in Chinese patients with Parkinson’s disease. *Brain, Behavior, and Immunity*.

[132] Chen S. J., Chen C. C., Liao H. Y. (2022). Association of fecal and plasma levels of short-chain fatty acids with gut microbiota and clinical severity in patients with Parkinson disease. *Neurology*.

[133] Dinan T. G., Cryan J. F. (2017). Gut instincts: microbiota as a key regulator of brain development, ageing and neurodegeneration. *The Journal of Physiology*.

[134] Dalile B., Van Oudenhove L., Vervliet B., Verbeke K. (2019). The role of short-chain fatty acids in microbiota-gut-brain communication. *Nature Reviews Gastroenterology and Hepatology*.

[135] Aho V. T. E., Houser M. C., Pereira P. B. (2021). Relationships of gut microbiota, short-chain fatty acids, inflammation, and the gut barrier in Parkinson’s disease. *Molecular Neurodegeneration*.

[136] Lujan J. A., Rugeles M. T., Taborda N. A. (2019). Contribution of the microbiota to intestinal homeostasis and its role in the pathogenesis of HIV-1 infection. *Current HIV Research*.

[137] Neff C. P., Krueger O., Xiong K. (2018). Fecal microbiota composition drives immune activation in HIV-infected individuals. *EBioMedicine*.

[138] Bowen L. N., Smith B., Reich D., Quezado M., Nath A. (2016). HIV-associated opportunistic CNS infections: pathophysiology, diagnosis and treatment. *Nature Reviews Neurology*.

[139] Siripurapu R., Ota Y. (2023). Human immunodeficiency virus: opportunistic infections and beyond. *Neuroimaging Clinics of North America*.

[140] Barrett E., Ross R. P., O’toole P. W., Fitzgerald G. F., Stanton C. (2012). *γ*-Aminobutyric acid production by culturable bacteria from the human intestine. *Journal of Applied Microbiology*.

[141] Holzer P., Farzi A. (2014). Neuropeptides and the microbiota-gut-brain axis. *Advances in Experimental Medicine and Biology*.

[142] Wang X., Sun G., Feng T. (2019). Sodium oligomannate therapeutically remodels gut microbiota and suppresses gut bacterial amino acids-shaped neuroinflammation to inhibit Alzheimer’s disease progression. *Cell Research*.

[143] Xiao S., Chan P., Wang T. (2021). A 36-week multicenter, randomized, double-blind, placebo-controlled, parallel-group, phase 3 clinical trial of sodium oligomannate for mild-to-moderate Alzheimer’s dementia. *Alzheimer’s Research and Therapy*.

[144] Wang X., Zhang P., Zhang X. (2021). Probiotics regulate gut microbiota: an effective method to improve immunity. *Molecules*.

[145] Gori A., Rizzardini G., Van’t Land B. (2011). Specific prebiotics modulate gut microbiota and immune activation in HAART-naive HIV-infected adults: results of the “COPA” pilot randomized trial. *Mucosal Immunology*.

[146] Yang W., Yu T., Huang X. (2020). Intestinal microbiota-derived short-chain fatty acids regulation of immune cell IL-22 production and gut immunity. *Nature Communications*.

[147] Liu J., Chen F., Wang X., Peng H., Zhang H., Wang K. J. (2020). The synergistic effect of mud crab antimicrobial peptides sphistin and sph(12-38) with antibiotics azithromycin and rifampicin enhances bactericidal activity against Pseudomonas aeruginosa. *Frontiers in Cellular and Infection Microbiology*.

[148] Korpela K., Salonen A., Virta L. J. (2016). Intestinal microbiome is related to lifetime antibiotic use in Finnish pre-school children. *Nature Communications*.

[149] Qian X., Yanagi K., Kane A. V. (2020). Ridinilazole, a narrow spectrum antibiotic for treatment of Clostridioides difficile infection, enhances preservation of microbiota-dependent bile acids. *American Journal of Physiology-Gastrointestinal and Liver Physiology*.

[150] Moura I. B., Grada A., Spittal W. (2022). Profiling the effects of systemic antibiotics for acne, including the narrow-spectrum antibiotic sarecycline, on the human gut microbiota. *Frontiers in Microbiology*.

[151] Huang Y. S., Yang J. J., Lee N. Y. (2017). Treatment of Pneumocystis jirovecii pneumonia in HIV-infected patients: a review. *Expert Review of Anti-infective Therapy*.

[152] Akagawa Y., Kimata T., Akagawa S. (2020). Impact of long-term low dose antibiotic prophylaxis on gut microbiota in children. *The Journal of Urology*.

[153] Duan Y., Llorente C., Lang S. (2019). Bacteriophage targeting of gut bacterium attenuates alcoholic liver disease. *Nature*.

[154] Sinha A., Li Y., Mirzaei M. K. (2022). Transplantation of bacteriophages from ulcerative colitis patients shifts the gut bacteriome and exacerbates the severity of DSS colitis. *Microbiome*.

[155] Federici S., Kredo-Russo S., Valdes-Mas R. (2022). Targeted suppression of human IBD-associated gut microbiota commensals by phage consortia for treatment of intestinal inflammation. *Cell*.

[156] Wastyk H. C., Fragiadakis G. K., Perelman D. (2021). Gut-microbiota-targeted diets modulate human immune status. *Cell*.

[157] Kong C., Yan X., Liu Y. (2021). Ketogenic diet alleviates colitis by reduction of colonic group 3 innate lymphoid cells through altering gut microbiome. *Signal Transduction and Targeted Therapy*.

[158] Sainz T., Diaz L., Rojo D. (2022). Targeting the gut microbiota of vertically HIV-infected children to decrease inflammation and immunoactivation: a pilot clinical trial. *Nutrients*.

[159] Klatt N. R., Broedlow C., Osborn J. M. (2021). Effects of persistent modulation of intestinal microbiota on SIV/HIV vaccination in rhesus macaques. *NPJ Vaccines*.

